# Efficiency of Gum Rosin-Coated Personal Protective Clothing to Protect against Chlorpyrifos Exposure in Applicators

**DOI:** 10.3390/ijerph19052594

**Published:** 2022-02-23

**Authors:** Ratana Sapbamrer, Manoch Naksata, Surat Hongsibsong, Jiraporn Chittrakul, Wilawan Chaiut

**Affiliations:** 1 Department of Community Medicine, Faculty of Medicine, Chiang Mai University, 110 Inthavaroros Road, Sri Phum Subdistrict, Muang District, Chiang Mai 50200, Thailand; jerasooutch@gmail.com (J.C.); wilawan_chai@cmu.ac.th (W.C.); 2 Department of Physics and Material Science, Faculty of Science, Chiang Mai University, 239, Huay Kaew Road, Suthep Subdistrict, Muang District, Chiang Mai 50200, Thailand; manochnak@gmail.com; 3 School of Health Sciences Research, Research Institute for Health Sciences, Chiang Mai University, 110 Inthavaroros Road, Sriphum Subdistrict, Muang District, Chiang Mai 50200, Thailand; s_hongsibsong@hotmail.com

**Keywords:** personal protective equipment, PPE, pesticide, insecticide, chlorpyrifos, farmer, clothing

## Abstract

An ideal theoretical model for personal protective clothing (PPC) is to protect wearers from external workplace hazards while meeting certain ergonomic requirements. This study aims to compare the efficiency of different personal protective clothing in its protection against chlorpyrifos, including gum rosin-coated PPC, commercial PPC (Tychem^®^ coverall), and everyday clothing, during application under field conditions. Perception of discomfort and physiological effects after wearing PPC were also investigated. Thirty-one applicators were invited and consented to participate in the study. The study established that the median percentage of chlorpyrifos protection efficiency was 90.7% for commercial PPC, 89.2% for gum rosin-coated PPC, and 76.5% for everyday PPC. When the protection efficiency was compared among the different types of PPC, the percentage protection efficiency of gum rosin-coated PPC was not significantly different from that of commercial PPC. The percentage protection efficiencies of commercial PPC and gum rosin-coated PPC were significantly higher than that of everyday PPC. The major characteristics of gum rosin-coated PPC were water-repellency, breathability, low cost, and ease of manufacture. Therefore, this study suggests that gum rosin-coated PPC is a reasonable alternative PPC for farmers, particularly in low and middle-low-income countries and tropical climates, to protect against pesticides while providing acceptable comfort.

## 1. Introduction

Pesticides are used in farming for increasing crop productivity and protecting crops from pests. However, occupational exposure to pesticides in agriculture is a major risk factor in the development of acute and chronic health effects. These health effects include eczema, asthma, neurological diseases, reproductive diseases, and cancers [1,2]. Occupational exposure to pesticides often occurs when pesticide handlers apply, mix, load, and transport pesticides. The most common routes of exposure to pesticides are dermal and via inhalation. Dermal exposure often occurs when pesticide handlers wear an inappropriate type of personal protective equipment (PPE) during application. Therefore, wearing appropriate PPE while handling pesticides could reduce pesticide exposure [3,4].

The most common PPE worn by pesticide handlers globally was long-sleeved shirt (66.1%), long-sleeved trousers (71.1%), and a hat (47.3%), while an apron (8.6%), goggles (24.3%), and gloves (40.5%) were used the least [5]. Although most pesticide handlers wore long-sleeved shirts and long trousers to protect themselves from dermal exposure to pesticides, these items of personal protective clothing (PPC) did not meet the standard for chemical protection. This PPC is usually made of woven fabric, therefore pesticides can pass through the skin, depending on the type of pesticide and the type and weight of the fabric [6,7]. Commercial PPC has proven to meet the standard for protection against chemicals, but pesticide handlers in low and middle-low-income countries cannot purchase commercial PPC because it is relatively expensive and is usually single-use (disposable). Commercial PPC is also often made of non-porous materials which cause mental strain, discomfort, and heat stress while working in hot and humid conditions [8,9]. It is therefore impractical for working conditions in tropical countries, including Thailand.

Chlorpyrifos is one of the most widely used organophosphate insecticides in agriculture and has been detected in vegetables and fruits globally [10,11]. Chlorpyrifos can pose a great risk to environments, animals, and human health [11]. Scientific evidence in humans found an association between exposure to chlorpyrifos and neurological and neurodevelopmental disorders [12,13]. In Thailand, chlorpyrifos was extensively and unlimitedly used for agriculture over recent decades until 2020, when Thailand revised its rules and regulations to restrict chlorpyrifos use. Farmers must now adhere to the following rules and regulations: (1) register as a farmer with the Department of Agricultural Extension; (2) attend training offered by Department of Agricultural Extension every 3 years and pass a test; (3) show an identity card before purchasing chlorpyrifos; (4) only use chlorpyrifos for fruit trees, flower trees, and dry crops; (5) apply chlorpyrifos with pesticide sprayers and wear PPE during application [14,15]. Although restrictions have been applied to the use of chlorpyrifos, farmers still experience potential risks from exposure to chlorpyrifos due to inappropriate PPC. They usually wear everyday clothing made of woven fabric during pesticide application because of the low cost and breathability of this PPC option [16].

PPC coated with gum rosin was developed by Naksata et al. [17]. Denim cotton fabric was coated with gum rosin solution to provide water repellent properties and the protective efficiency against chlorpyrifos was investigated in a laboratory (a closed chamber). This protective efficiency ranged between 99.85–99.97%, while the protective efficiency of Tychem^®^ C coverall (commercial PPC) was 99.95%. When comparing the protective efficiency of the gum rosin-coated PPC and the commercial PPC, this was not significantly different. We expected that the protective efficiency of the gum rosin-coated PPC would not differ from that of commercial PPC. We also expected that gum rosin-coated PPC would, at the same time, feel comfortable while working in hot and humid conditions. Therefore, this study aims to compare the efficiency of different personal protective clothing in protection against chlorpyrifos, including gum rosin-coated PPC, commercial PPC (Tychem^®^ coverall), and everyday clothing during application under field conditions. Perception of discomfort and physiological effects after wearing PPC were also investigated.

## 2. Materials and Methods

### 2.1. Personal Protective Clothing (PPC)

**Figure 1:**
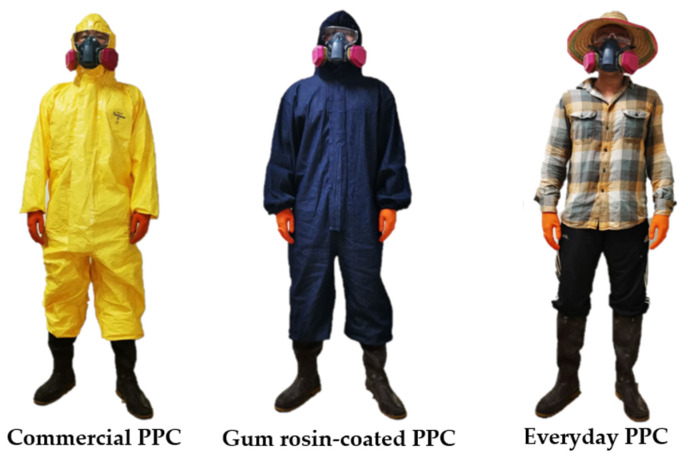
Three types of personal protective clothing (PPC).

Three types of PPC were tested in the study, including commercial PPC, gum rosin-coated PPC, and everyday clothing. The Tychem^®^ coverall, a commercial PPC manufactured by DuPont, was chosen to represent a commercial PPC for protection against pesticides. The Tychem^®^ coverall is designed to protect workers from intensive sprays and splashes of hazardous substances as well as very fine particles. It also complies with European standards for limited-use chemical protective clothing. The repellency index ranged from 92.6% for isopropanol to 99.7% for 10% sodium hydroxide. Gum rosin-coated PPC was developed by Naksata et al. [17]. Denim cotton fabric was chosen to be coated with gum rosin solution because it provided the best level of pesticide protection in a preliminary laboratory test. Denim cotton fabric was made into a coverall with the same size and design as the Tychem^®^ coverall, and then coated with gum rosin. The coating process was carried out in accordance with the method described by Naksata and Naksata [18] (Petty Patent no. 7450, 8 July 2016, Thailand) as follows: (1) PPC laundered with detergent and tap water at 60–80 °C; (2) soaked with gum rosin solution 1–3% *w*/*v* (Chemwinfo Co. Ltd., Bangkok, Thailand) for 15 min; (3) soaked with potassium aluminum sulfate 1–3 *w*/*v* (World Chemical Co., Ltd., Chiang Mai, Thailand) for 15 min; (4) spin-dried at 300 rpm for 3 min; and (5) dried at 60–80 °C. Everyday clothing that applicators usually wore during pesticide application included a long-sleeved shirt, long-sleeved trousers, and a hat. This equipment is generally made of woven fabric (Figure 1).

To protect the applicators from exposure to pesticides via other routes, respirators with cartridges, goggles, rubber gloves, and rubber boots were provided during application. In addition, clean clothes (long-sleeved shirt and long-sleeved trousers) were worn inside before wearing the tested PPC, in order to minimize exposure to pesticides.

### 2.2. Study Site and Participants

During October and November 2021, the research was conducted on a farm in Chiang Mai, northern Thailand. Thirty-one applicators were invited and consented to participate in the study. The participants all had the following characteristics: aged more than 18 years old, healthy, usually applied chlorpyrifos on their farm, diluted chlorpyrifos for application according to the recommendations on the pesticide’s label (approximately 20 mL/10 L of water), and used a motor knapsack spray machine with cone nozzle for the application. Participants could apply any brand of chlorpyrifos that was formulated as 40% *w*/*v* emulsifiable concentrate and was usually applied on their farm. The participants were asked to specify the date and time of the chlorpyrifos spraying on their farms, and they were given an appointment for the investigation schedule.

The three types of PPC were tested by each participant, with only one tested each day, thus each participant required three days for investigation. In each PPC test, the participant sprayed chlorpyrifos into the field for 20 min without stopping. To control environmental and field conditions, each PPC was tested at the same time of day (in the morning), for the same amount of time (20 min), on the same crops, and with the same spraying machine.

### 2.3. Measurement Climatic Conditions in the Field

To control the environmental field conditions among the tested PPC, climatic conditions, including temperature (Elitech BT-3 thermometer, San Jose, CA, USA), relative humidity (Elitech BT-3 thermometer, CA, USA), and wind velocity (UNI-T UT363 Digital Wind speed Meter, Dongguan, Guangdong, China) were measured during the investigation. The climatic conditions were not significantly different during the test period (*p* > 0.05) (Table 1).

### 2.4. Measurement of Protective Efficiency of PPC

Dermal exposure was measured using the pads technique. Before the participants wore the PPC, clean clothes (long-sleeved shirt and long-sleeved trousers) were worn underneath to minimize exposure to pesticides. The inside clothes were representative of the participant’s body size. Internal pads were then attached to the inside clothes. Alpha-cellulose pads (diameter 4.4 cm) backed with aluminum foil were attached to the following 10 locations of the body; head, neck, chest, upper arm, forearm, back, belly, genital area, upper leg, and lower leg. Internal pads were representative of potential dermal exposure (PDE), i.e., amounts of chlorpyrifos pass through the PPC and contacted the skin. External pads were attached to the PPC at the same positions as the internal pads. External pads were representative of actual dermal exposure (ADE), i.e., amounts of chlorpyrifos contacted uncovered skin. At the end of spraying, the pads were removed and stored at −20 °C until chemical analysis.

The average total body surface area was approximately 20,400 cm^2^, and the total surface area of ten pads was approximately 694.26 cm^2^ [19]. PDE and ADE of total body surface areas were calculated by multiplying amounts of chlorpyrifos in total pads by 29.38. Protective efficiency against chlorpyrifos is as follows:

% protective efficiency = ((ADE − PDE) × 100)/ADE

where ADE = total amounts of chlorpyrifos from external pads; PDE = total amounts of chlorpyrifos from internal pads

### 2.5. Analysis of Chlorpyrifos

Chlorpyrifos (CAS Number: 2921-88-2) was purchased from Dr. Ehrenstorfer GmbH (Augsburg, Germany). The pad samples were extracted and analyzed by using the method described by Sapbamrer and Hongsibsong [20]. Twenty mL of acetonitrile (HPLC grade, J.T. Baker, Phillipsburg, NJ, USA) was used to extract the pad sample, which was agitated for 5 min. With 20 mL and 10 mL of acetonitrile, the extraction was performed twice more. To remove water from the extract solution, 3 g of magnesium sulfate (analytical grade, Fluka, Buchs, Germany) and sodium chloride (analytical grade, Fluka, Buchs, Germany) were added. The solution was filtered through filter paper containing 2 g of anhydrous sodium sulfate (analytical grade, Fluka, Buchs, Germany), and then evaporated at 40 °C until dry. The evaporation flask was rinsed with 5 mL ethyl acetate (HPLC grade, J.T. Baker, Phillipsburg, NJ, USA) and the nitrogen dried. Finally, using a 0.25 μm syringe filter, the residue was reconstituted in 1 mL of ethyl acetate. Gas chromatography (Hewlett-Packard 7890 Series, Palo Alto, CA, USA) with a flame photometric detector (FPD) and a capillary column DB-1701 (14 percent cyanopropyl-phenyl-methyl-poly-siloxane column −0.25 mm. I.D. × 30 m length × 0.25 m film thickness) was used for chlorpyrifos analysis. The injection port was set to 250 °C (spitless mode) and the detector to 250 °C. The limit of detection (LOD) was 0.0020 μg/L, and the limit of quantification (LOQ) was 0.020 μg/L. Recoveries were 99.6% for intra-batch and 76.93% for inter-batch. Relative SD coefficient (%RSD) was 1.40% for intra-batch and 6.48% for inter-batch.

### 2.6. Measurement of Physiological Effect and Perception of Discomfort

Tympanic temperature (EasyTherm, infrared thermometer, Shenzhen, China), pulse rate, and blood oxygen levels (YX102 oximeter, Yuwell, Danyang, China) were measured before and after the investigation. At the end of the application, participants were asked about their perception of discomfort. Six parameters of perception were as follows: heat, humidity, itchiness, inconvenience at work, feeling unfit, and fatigue. These questions were asked by a rating scale, ranging from 1 to 7, with 1 representing “comfortable”, 4 representing “acceptable”, and 7 representing “uncomfortable”.

### 2.7. Data Analysis

Descriptive statistics, including geometric mean, median, 95% confidence interval (95%CI), the 25th percentile and the 75th percentile, were presented. Normal distribution of data was tested before analyzing inferential statistics, and Kruskal-Wallis test was used to compare the efficiency to protect against chlorpyrifos among the tested PPC. Pairwise comparison was analyzed by using Dwass-Steel-Critchlow-Fligner. One-way ANOVA was used to compare the environmental conditions and the perception of discomfort among the tested PPC. A paired t-test was used to compare physiological effects before and after spraying pesticides. A *p* value less than 0.05 was statistically significant. The significance of *p* < 0.05 was indicated by *, and *p* < 0.01 was indicated by **.

## 3. Results

### 3.1. Actual Dermal Exposure (ADE) and Potential Dermal Exposure (PDE) for the Total Surface Body of Applicators

The median ADE of chlorpyrifos was 13,632 μg for commercial PPC, 10,547 μg for gum rosin-coated PPC, and 15,513 μg for everyday PPC. The median PDE of chlorpyrifos was 1058 μg for commercial PPC, 1028 μg for gum rosin-coated PPC, and 2556 μg for everyday PPC (Table 2).

### 3.2. Comparison of Chlorpyrifos Protection Efficiency among Tested PPC

The median percentage of chlorpyrifos protection efficiency was 90.7% for commercial PPC, 89.2% for gum rosin-coated PPC, and 76.5% for everyday PPC. When the protection efficiency among tested PPC was compared, the % protection efficiency of gum rosin-coated PPC was not significantly different from that of commercial PPC. The % protection efficiencies of commercial PPC and gum rosin-coated PPC were significantly higher than those of everyday PPC (Table 2).

### 3.3. Physiological Effects among Tested PPC

**Figure 2:**
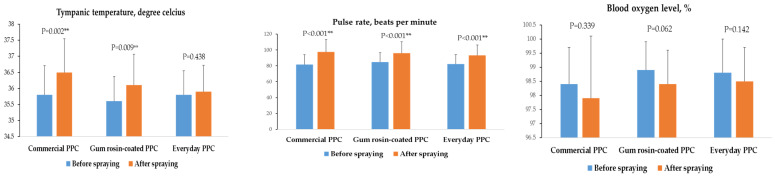
Physiological effects among tested PPC. ** *p* < 0.01.

Pulse rate and tympanic temperature after spraying chlorpyrifos for all tested PPC were significantly higher than before spraying chlorpyrifos. However, blood oxygen level for all tested PPC was not significantly different (Figure 2).

### 3.4. The Perception of Discomfort among Tested PPC

**Figure 3:**
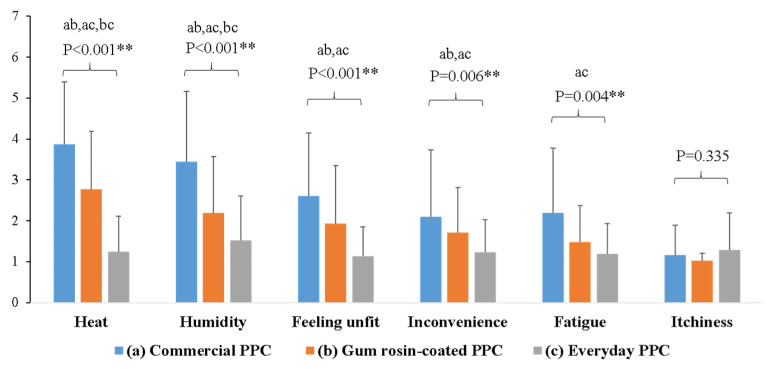
Perception of discomfort among tested PPC. ** *p* < 0.01. ab the parameters for commercial PPC were different from those for gum rosin-coated PPC; ac the parameters for commercial PPC were different from those for everyday PPC; bc the parameters for gum rosin-coated PPC were different from those for everyday PPC.

Commercial PPC had the highest perception of discomfort in all domains, particularly regarding heat and humidity. The commercial PPC had a significantly higher perception of discomfort than gum rosin-coated PPC and everyday PPC, and the gum rosin-coated PPC had a significantly higher perception of discomfort than the everyday PPC. The commercial PPC had a significantly higher feeling of unfitness and inconvenience for work than the gum rosin-coated PPC and the everyday PPC. Regarding fatigue, the commercial PPC had a significantly higher perception than the everyday PPC (Figure 3).

## 4. Discussion

This study found that the effectiveness of gum rosin-coated PCC in protecting against chlorpyrifos was comparable to that of commercial PPC. Gum rosin-coated PCC was manufactured from denim fabric and treated with gum rosin solution to provide water repellency. The results of the experiment by Naksata and Naksata (2016) showed that its water resistance lasted more than 24 h, and its contact angle of water repellency was greater than 120° [18]. The study by Sapbamrer et al. [6] investigated the efficiency of denim fabric for protection against chlorpyrifos in a laboratory (a gravimetric method) according to the ISO 22608 standard and compared the efficiency of denim and gum rosin-coated denim. The findings revealed that the uncoated gum resin denim had a protective efficiency of 60.8% and the gum rosin-coated denim had a protective efficiency of 93.6%. In addition, the study by Naksata et al. [17] developed gum rosin-coated PPC with five different types of fabric and investigated the efficiency of protection against chlorpyrifos in a laboratory (a closed chamber) according to the ASTM F1359/F1359M-16a (procedure A). The results found that the protective efficiency ranged from 99.8% to 99.9%, and gum rosin-coated denim had the highest protective efficiency. Therefore, these two laboratory studies confirmed that gum rosin-coated denim showed high efficiency regarding protection against chlorpyrifos.

When comparing the protective efficiency of gum rosin-coated PPC with commercial PPC in other studies under field conditions, the protective efficiencies were similar, ranging from 97% to 98.7% [21,22,23]. When comparing the protective efficiency of gum rosin-coated PPC with woven fabrics with repellent finishes, the protective efficiencies were also similar, ranging from 95.8 to 97.1% [24] (Table 3). A study by Rahman Bhuiyan et al. [25] also found the efficiency of polyurethane-aerogel finished fabrics in protecting against chemicals to be 100%. In addition, a study by Shaw and Schiffelbein [26] evaluated 100 fabrics that were suitable for applying pesticides, and the study claimed that woven fabrics with repellent finishes had a mean percentage penetration ≤ 5%. However, as these studies investigated or tested different chemicals, the interpretation of the findings should be considered. As a result of this study, it is suggested that gum rosin-coated PPC should be the choice of personal protective equipment for applicators (Table 3).

The results of this study also demonstrated that everyday PPC had the least efficiency in protecting against chlorpyrifos. The results agreed with a study of Aprea et al. [4] who suggested that cotton garments had less skin protection than waterproof garments and Tyvek coveralls. Most applicators wore long-sleeved shirt and long-sleeved trousers which were generally made of cotton-woven fabric to protect themselves from dermal exposure to pesticides. Therefore, some pesticides could penetrate through skin, depending on the types of pesticides and the characteristics of fabrics. Previous studies have indicated that types of fabric, weight, thickness, yarn twist, and wicking were all factors affecting pesticide penetration [6,27] Therefore, wearing everyday PPC for protecting against pesticides should be considered as unacceptable.

Wearing PPC may have negative physiological effects and cause discomfort. This study identified that tympanic temperature was significantly higher after wearing all types of PPC during application compared with before wearing, whereas pulse rate was significantly higher after wearing commercial and gum rosin-coated PPC. These findings supported the findings of Coca et al. [28] who found that wearing PPE increased core temperature at the end of exercise intervention but did not change the heart rate. In addition, a study by de Almeida et al. [29] suggested that wearing PPE increased body temperature. It took 15 min to raise 1 °C of body temperature, compared to 40 min without PPE. In terms of the level of perceived discomfort, this study found that gum rosin-coated PPC led to a perception of discomfort in measures of heat, humidity, and feeling unfit lower than those while wearing commercial PPC. However, the gum rosin-coated PPC had higher perceived levels of heat, feeling unfit, and inconvenience than when wearing everyday PPC. Importantly, the commercial PPC had the highest perception of discomfort in all domains, except itchiness, when compared with other PPC. An ideal theoretical model of PPC is that it protects wearers from external workplace hazards while meeting certain ergonomic requirements. It should not aggravate discomfort and physiological strain while still being comfortable, affordable, and practical in working conditions. However, most PPC with high degrees of protection generally interferes with heat exchange by perspiration evaporation due to its impermeability to fluids [9,30]. Furthermore, most available studies have focused on the effectiveness of PPC for protecting against hazards, and the effectiveness of PPC in working conditions may be overestimated due to the complexities of real-life practical conditions. In addition, wearing PPC can be impractical due to several factors, including thermic and mechanical discomfort, as well as being quite expensive, difficult to obtain, and causing restricted movement [30,31].

Wearing PPC, particularly in hot and humid environments, might cause a rise in body temperature, making the wearers feel hot, uncomfortable, and fatigued [32]. Occupational heat exposure might also raise the risk of injury [33]. A review by Garrigou et al. [31] clearly claimed that wearing PPE does not always provide effective protection and might also cause severe discomfort. Commercial PPC is made of non-woven synthetic materials treated with chemicals to make them non-porous and to protect against high level hazards [21,34]. Because of this property of commercial PPC, this type of PPC caused more heat exchange and discomfort than other types of PPC. The study by Davey et al. [35] also stated that wearing PPE made the task more difficult for workers. In addition, the costs of commercial PPE were prohibitively expensive for farmers, particularly in low and middle-low-income countries, therefore, most farmers cannot afford it [16]. A systematic review by Sapbamrer and Thammachai [5] also suggested that income status had effects on PPE use and pesticide safety practices. Farmers with a higher economic status were more likely to purchase PPE and had more options for higher-quality PPE [36,37]. Regarding gum rosin-coated PPC, the ultimate goal of gum rosin-coated fabric was to provide adequate pesticide protection and comfort under hot and humid conditions. Because of its porous and breathability property, this PPC can ventilate better than commercial PPC, resulting in reducing heat and humid accumulation, and being more comfortable. Even though everyday PPC caused the least discomfort, wearing it provided an unacceptable level of pesticide protection. Therefore, the results of this study suggest that gum rosin-coated PPC is the most appropriate alternative PPC for farmers, particularly those in low and middle-low-income countries and countries with tropical climates, to protect against pesticides while providing acceptable comfort.

This study was conducted in field conditions to control co-variables such as the farmer, farming, and environmental factors. However, some limitations should be considered to interpretation of the results. Firstly, the study was conducted using only chlorpyrifos, therefore surrogate chemicals of pesticides in accordance with EN/ISO 27068 are needed in further research. Furthermore, PPE products must comply with the EU Declaration of Conformity, according to the PPE Regulation (EU) 2016/425 before placement on the market. However, gum rosin-coated PPC did not concern the compliance with this EU regulation, and this study did not assess all protective parameters. As a result, more research on these issues is needed. Secondly, the study was conducted for the first time using these fabrics, and further studies should be conducted to test fabrics that have been washed 30 times in accordance with ISO27065. Thirdly, the measurement of the perception of discomfort was based on subjective questions; therefore, the interpretation of the results should be carefully considered. Finally, only the level of dermal exposure was measured as part of this study. However, urinary organophosphate metabolites have been found to be a good representation of potential dermal exposure [38]. Consequently, further research using biological biomarkers is warranted.

## 5. Conclusions

This study concluded that the effectiveness of gum rosin-coated PCC in protecting against chlorpyrifos was comparable to that of commercial PPC in field conditions. Wearing gum rosin-coated PPC also demonstrated a lower level of perceived discomfort than commercial PPC. As a result, gum rosin-coated PPC is a reasonable alternative PPC for insecticide protection for farmers in low and middle-low-income countries and tropical climates, and might be appropriate for small-scale businesses in rural areas to provide and sell in their communities. Strategies to protect applicators from pesticide exposure, to comply with PPE regulation, and to enable comfort require further research and innovation to achieve full effectiveness.

## 6. Patents

The coating process of PPC with gum rosin was carried out in accordance with the method described by Naksata M. and Naksata V. (Petty Patent no. 7450, 8 July 2016, Thailand). Naksata, M. is a co-author in this study.

## Figures and Tables

**Table 1:** Environmental conditions in the agricultural field (*n* = 31).

Parameter	Commercial PPC	Gum Rosin-Coated PPC	Everyday PPC	*p* Value
Temperature, °C	28.03 ± 1.84	27.45 ± 1.59	27.47 ± 1.04	0.240
Relative humidity, %	75.06 ± 7.51	75.52 ± 6.46	77.13 ± 3.22	0.368
Wind velocity, ft/min	88.19 ± 26.92	90.00 ± 26.03	85.65 ± 23.74	0.798

**Table 2:** Actual dermal exposure, potential dermal exposure, and protective efficiency of chlorpyrifos among tested PPC (*n* = 31).

Parameter	Commercial PPC ^a^		Gum Rosin-Coated PPC ^b^		Everyday PPC ^c^		*p* Value
	Geometric Mean (95%CI)	Median (25th–75th Percentile)	Geometric mean (95%CI)	Median (25th–75th Percentile)	Geometric Mean (95%CI)	Median (25th–75th Percentile)	
ADE (μg)	12,206 (9364–16,164)	13,632 (3731–52,884)	13,849 (11,651–16,355)	10,547 (5876–24,797)	13,066 (10,501–16,549)	15,513 (6140–24,385)	0.845
PDE (μg)	1143 (930–1418)	1058 (529–1822)	1298 (1078–1539)	1028 (617–2174)	2864 (2241–3712)	2552 (852–5788)	0.012 * ac,bc
% protection efficiency	81.9 (78.3–84.4)	90.7 (68.9–95.3)	84.3 (82.3–88.0)	89.2 (78.3–96.0)	64.9 (59.7–69.8)	76.5 (53.2–87.9)	0.002 ** ac,bc

Note: data was analyzed with Kruskal-Wallis test (multiple comparisons) and Dwass-Steel-Critchlow-Fligner (pairwise comparison). ADE = actual dermal exposure; PDE = potential dermal exposure; % protective efficiency = ((ADE − PDE) × 100)/ADE; 95%CI = 95% confidence interval of geometric mean; ^a^ commercial PPC; ^b^ gum rosin-coated PPC; ^c^ everyday PPC; ac the parameters for commercial PPC were different from those for everyday PPC; bc the parameters for gum ros-in-caoted PPC were different from those for everyday PPC; * *p* < 0.05; ** *p* < 0.01.

**Table 3:** Comparison of the protective efficiency of gum rosin-coated PPC with commercial PPC and other repellent finished PPC under laboratory and field conditions.

Condition	Type of PPC	Test Chemicals	% Protection Efficiency	Authors
**Laboratory Conditions**				
	**Commercial PPC**			
	Tychem^®^ coverall (closed chamber)	Chlorpyrifos	99.9% ^a^, 99.9% ^b^	Naksata et al. [17]
	**Repellent finished PPC**			
	Gum rosin-coated denim (closed chamber)	Chlorpyrifos	99.9% ^a^, 99.9% ^b^	Naksata et al. [17]
	Gum rosin-coated denim (gravimetric method)	Chlorpyrifos	93.6% ^a^	Sapbamrer et al. [6]
	Fluorocarbon finishes	Copper hydroxide	93.9–96.8% ^a^	Espanhol-Soares et al. [24]
**Field Conditions**				
	**Commercial PPC**			
	Tychem^®^ coverall	Chlorpyrifos	81.9% ^a^, 90.7% ^b^	The present study
	Category III Type-partial body gown	Spinosad	98.7% ^a^	Thouvenin et al. [21]
	Tyvek coverall	azinphos-methyl, terbuthylazine, alachlor, dimethoate, and dicamba	>97% ^a^	Protano and Guidotti [22]
	Tyvek coverall	azinphos-methyl, terbuthylazine, alachlor, dimethoate, and dicamba	97.6% ^a^	Vitali et al. [23]
	**Repellent finished PPC**			
	Gum rosin-coated PPC	Chlorpyrifos	84.3% ^a^, 89.2% ^b^	The present study
	Fluorocarbon finishes	Sulfate manganese	95.8–97.1% ^a^	Espanhol-Soares et al. [24]

^a^ presented as mean or geometric mean; ^b^ presented as median.

## Data Availability

Not applicable.
